# Foreign

**Published:** 1841-08-07

**Authors:** 


### FOREIGN.

*On the Diagnosis of Abdominal Inflammations.* By William Griffin, M. D., M. R. C. S. E., Physician to the county of Limerick Infirmary, &c.—[This paper contains some new and striking illustrations of the important practical fact to which Dr. Griffin had previously called attention, viz., that inflammatory, or other affections of the spinal cord, or of its nerves, at their origin, more frequently simulate abdominal and thoracic inflammations, and are more frequently mistaken and maltreated, than is at all imagined.

We have not room to give the cases, but extract the greater part of the general observations.]

There are three effects very common to inflammation, or disorder in the spinal cord, or at the trunks of its nerves: 1st, Superficial tenderness, more or less exquisite, and either limited to the integument immediately over or about the affected portion of the cord, or extending thence to the front of the abdomen, or thorax, in the direction of the spinal nerves; or occupying the whole cutanous surface of those parts of the body, which are below the portion of the cord affected. 2dly, Pain either close to the affected portion of the cord, or at the extremities of the nerves, which have their origin there; or in the ganglionic nerves supplying the viscera, which have connection with that portion of the cord. 3dly, Loss of power evinced in partial or complete palsy of the parts or organs, to which the affected nerves are distributed. These effects often occur simultaneously, but any one of them may also occur independently of the existence of the others, offering very strong evidence that the sensibility of the surface or skin and that which exists in internal organs is dependent upon nerves, which, though sentient, are as distinct from one another as they are from those on which the power of motion depends. Keeping these ordinary effects of disorder of the spinal cord in view, it must appear obvious, when we detect soreness or tenderness on pressure in the region of the liver or spleen, or of the lower abdominal viscera, how important and essential it is for us to ascertain whether the soreness be superficial or deep-seated, which we can almost always do by careful examination. Again, when pain is complained of in the region of the liver, or middle or lower parts of the abdomen, how necessary is it to ascertain, whether, as in the case of soreness, it be superficial; and, if deep-seated, whether it be merely an affection of the nerves of the part, and probably connected with some affection of the adjoining portion of the spinal cord, or whether the internal organ itself be in a state of acute or chronic inflammation. Finally, if there be oppression in breathing, whether it arises from deficient action in the respiratory nerves, and, as a consequence, imperfect action of the respiratory muscles, or imperfect performance of the process of oxygenation of the blood in the lungs; or from actual inflammation, or organic disease of the mucous membrane, or parenchyma of these organs. Or if there be obstinate constipation of bowels, whether it depends on spasm, or enteric inflammation; or whether purely or deficient power, or partial palsy of the muscular fibres of the intestines.

In determining the diagnosis of abdominal inflammation, where both pain and tenderness on pressure exist, we should always endeavour to ascertain:

1st. Whether there be any pain or tenderness on pressure in the corresponding portion of the spinal column; because, if there be, although it may not absolutely decide whether inflammation be present or not, it is quite sufficient to account for both the pain and tenderness, without assuming the existence of any inflammation.

2d. Whether there be no spinal tenderness or pain, the soreness of the abdomen be superficial or deep-seated, which may be ascertained with tolerable certainty in all cases by an examination directed to that end. And whether, if both superficial and deep-seated, as it usually is, in peritoneal inflammation, gentle, steady pressure with the flat of the hand can be easier borne, than with the points of the fingers. In pain and sorenes from affection of the spinal nerves it commonly can be so borne, while in peritonitis every kind of pressure, and even the weight of the bed-clothes is very distressing.

3d. Whether the boundaries of the pain or soreness extend beyond what the suspected inflammation could produce. Thus, if inflammation of the liver be suspected, and we find the soreness extending to the spine of the ilium, or groin, or to the opposite side of the abdomen to which the liver does not extend, it is obvious, the soreness cannot be attributable to mere disease of that organ. Again, if the whole abdomen be tender to the touch in a case otherwise closely resembling peritonitis, and we find the tenderness is not confined to the abdomen, but extends over the hips and lower extremities, it is obvious, we can attach no importance to the abdominal soreness as a sign of inflammation.

Finally, it should be recollected that constipation may depend on mere loss of power in the intestinal nerves, as well as on the spasm, obstruction, or inflammation, as the treatment in each case must necessarily be modified or directed by the supposed cause of this symptom.—*British and Foreign Med. Review, from Dublin Journal.*

---

*A Sketch of the character of* JAMES HOPE, M. D., F. R. S., *late one of the Physicians to St. George's Hospital, with brief preliminary notices of his Life.*—James Hope, the ninth child of Thomas Hope, Esq., of Prestbury Hall, Cheshire, was born at Stockport on the 23d of February, 1801. Very early indications of quickness and intelligence, of general activity, and of a mild, gentle disposition, gave a pleasing promise of future excellence.

Under an inefficient instructor, he was left, until nearly 11 years of age, to pursue in a great degree his own inclinations in reading. His avidity for knowledge, however, led him to turn over his father's books with intense interest, and to select for his perusal not only the productions of fancy and amusement, but also works of solid instruction and even of high genius. When about eight years old, Milton's Paradise Lost engaged his attention, as also Parke's Chemical Catechism, on which he happened to stumble. He became so engrossed with chemistry as at once to commence a zealous experimentalist, and was not a little mortified, after many trials, to find himself unable to make gunpowder for the supply of his sporting brothers.

From the age of eleven to seventeen and a half, he made steady advances in his classical studies under able masters; and having determined, if possible, to be a first-class man at college, he did not relax his vigorous application until he had mastered the highest Greek authors. During this period he was also storing his mind with general literature, and becoming well acquainted with works of taste.

Having a great desire to practise at the English bar, when the period for his leaving school arrived he was much disappointed to find that his father had neglected to secure him a place at Oxford, and was bent on his entering on mercantile pursuits. This Dr. Hope positively refused to do, and after a year's delay a compromise was made. His father offered him the medical profession, and though he had a strong repugnance to it he consented on condition that he should be permitted to practise in London. Whilst waiting for rooms, he resided at Oxford for a year and a half. His father, tired of this delay, insisted upon his commencing his medical education, and, being thwarted in his cherished wish of completing his studies at Oxford, he proceeded to Edinburgh. At that celebrated school of medicine he pursued a laborious and highly successful course of study during five years; graduating with much eclat, and during the latter portion of the time holding, with signal practical advantage to himself and others, the offices of house-physician and house-surgeon at the Royal Infirmary.

From Edinburgh he went to London, with a view more especially to perfect his acquaintance with practical surgery. After diligently spending six months at St. Bartholomew's Hospital, he passed the College of Surgeons, and then commenced the tour of Europe. Attracted by some most favourable opportunities of cultivating auscultation and pathological science in Paris, he tarried in that capital an entire year. Thence he visited the principal cities and other interesting localities of Switzerland and Italy, and returned to England after an absence of two years. In December, 1828, Dr. Hope fixed himself as a physician in Lower Seymour street, Portman square.

In 1831 he married Anne, daughter of J. W. Fulton, Esq., of Upper Harley street, formerly of Calcutta. One child (a son) resulting from that happy union survives his lamented father. The same eventful year was marked by his election as senior physician to the Marylebone Infirmary, which office he held three years, only relinquishing it on his appointment as assistant physician at St. George's Hospital. That labourious field of exertion proved overwhelming. In the course of five years he saw 20,000, and took notes of 15,000 out patients, which he had there attended.

In 1839, on the resignation of Dr. Chambers, he was chosen physician to that hospital; but his exhausting duties in his former capacity had broken down the powers of his constitution. Phthisis supervened. At the age of forty, and on the 13th of May last, this amiable and accomplished physician departed from our world. In 1832 Dr. Hope was made a fellow of the Royal Society. In the succeeding years he received diplomas from several foreign societies, and in 1840 the College of Physicians elected him a fellow of their body.

By his will he directed that a post-mortem examination should be made, and that the persons employed should be at liberty to make any use of the morbid appearances they might think fit for the benefit of science, assigning as his reason that a physician should set a good example. The examination was accordingly made under the direction of Drs. Latham and Watson, who during his long illness had attended him with an unremitted attention, a sympathy, and a kindness, honourable alike to themselves and to their profession.

We proceed to give an outline of Dr. Hope's character, as viewed in its intellectual, moral, and professional aspects.

Dr. Hope's intellectual qualities were of a high order. A quick perception, a ready and singularly retentive memory, clearness, vigour, and comprehensiveness of thought, were happily associated with a sound and discriminating judgment, great decision, and a modest independence of mind.

From an early period Dr. Hope evinced a power of giving close and continuous attention to any subject in which he was engaged. Hence the impressions made upon his mind were singularly clear, and his recollections proportionally vivid and accurate.

*Decision of mind* was strikingly characteristic, especially as viewed in connection with that modest opinion of himself which accompanied him through life.

The *vigorous activity* of Dr. Hope's mind rendered occupation always agreeable. He loved information for its own sake, and scarcely less for the pleasure he found in its pursuit: and, having proposed to himself a high standard of attainment, he was never satisfied with any degree of excellence short of that which he believed himself able to reach.

*Comprehensiveness* was equally an attribute of Dr. Hope's mind. With an aptitude for observing the details of science he combined an admirable facility in classifying his facts, estimating their absolute and relative value, and cautiously deducing from them general principles.

He was also distinguished by *logical acumen.* Being himself a fair and close reasoner, never descending to sophistry or equivocation, he readily detected any fallacy in argument; while his thorough acquaintance with the laws of evidence led him to require the most complete proof of which the subject in question was susceptible.

*Versatility*, moreover, was happily associated in Dr. Hope's mental constitution, with his power of abstraction. Such was the elasticity of his mind that he could immediately rise, as with a bound, from the gravest to the most cheerful subject. During an extended classical education, and while studying the highest Greek authors, he was equally distinguished on the play-ground as the champion of athletic games; and subsequently, when zealously devoting himself to professional studies, his natural talent for drawing was so happily cultivated in the short intervals of relaxation as to enable him to copy, amongst others, a small landscape of Vanderveld with a spirit and fidelity worthy of an accomplished artist. This production of his pencil has a place in the valuable collection of the Lord President Hope.

Having thus cursorily noticed the particular features of Dr. Hope's intellectual character, it is but right to advert to the *singularly happy proportion* which subsisted between its several faculties, of which no one seemed to overpower or eclipse another. On the contrary, each appeared to sustain and invigorate the rest; and the harmonious working of the mind remarkably corresponded with the symmetrical arrangement of all its parts. Unity of design and consistency of action pervaded Dr. Hope's proceedings. His plans were wisely conceived, and as judiciously matured. At the fitting season they were carried into effect with determined perseverance, however obstructed by difficulties or delayed by disappointments. Nor could he be induced to swerve from his fixed purpose by the lure of pecuniary advantage or the prospect of an ephemeral fame.

In its *moral* aspect Dr. Hope's character appears to great advantage.

The mild benignity of his countenance, blended as it was with an expression of acute intelligence, gave a true indication of the liveliness of his feelings, and the kindness of his heart. His benevolence indeed would have frequently led him into extremes, had he not, in the exercise of a wise self-discipline, accustomed his feelings to the due control of his judgment.

That Dr. Hope was ambitious, cannot be denied; but his ambition was of the highest kind. He was ambitious of excellence, and was far more anxious *to be* good and great than to be thought so; and such was the noble elevation of his character that if undeserved distinctions had been within his reach he would have rejected them with disdain. To rise by legitimate means was doubtless his desire, but to rise in appearance only, or by the depression of others, was as foreign to the generosity of his nature as to his established principles of rectitude and honour.

Simplicity, ingenuousness, and magnanimity were strikingly manifested in his spirit and demeanour.

In the early stages of his career he was accustomed to view human nature in too favourable a light, an error not unfrequently attaching to young persons of high moral worth; and even when, by painful experience, he gained a deeper insight into the selfishness, double-mindedness, and envy which, alas! too often lurk even under a fair and polished exterior, he still endeavoured to put the kindest construction which truth would admit on human motives and actions. The tortuous policy of other men produced no change in him; his integrity sustained him. The directness and stability of his principles never allowed him to turn aside from the plain path of honour, even to secure the most important or favorite object.—He knew himself too well to imagine that any advantage unworthily obtained could yield him true or lasting satisfaction.

Yet such was the kindness of his spirit, and so exquisitely keen his sense of fairness and justice, that the ungenerous treatment which he sometimes received even from those from whom he had deserved better things, pierced him to the quick. Yet under such trying circumstances his magnanimity ever rose superior to all other considerations, and forbade him to retaliate on the offending party.

Dr. Hope was moreover strictly conscientious. An inflexible regard to truth and justice pervaded his whole conduct. He considered himself as constantly under the eye of Omniscience, and as responsible for the right improvement of his time, talents, property, and influence. In short he was a Christian, in the highest sense of the term, not from education merely, but after full and deliberate investigation. The momentous subject of religion demanded, he was aware, his deepest attention. Accordingly, he brought to its examination the same concentration of effort, the same patient scrutiny, the same logical acuteness, the same determination to believe nothing without conclusive evidence, which had proved availing in all his scientific researches. The result was a firm and abiding conviction of the Divine origin of Christianity, as revealed in the sacred records. Thus convinced, he unhesitatingly bowed to the authority of inspiration, and implicitly received its holy doctrines as the foundation of his highest hopes, and its holy precepts as the invariable rule of his conduct.—Nor is it superfluous to add, that his confidence in the religion of the Gospel remained unshaken to the last. It proved his highest solace in affliction, and his support in death.

The *professional* character of Dr. Hope strikingly exemplified the practical influence of the valuable qualities already specified.

The profession of physic was not in the first instance his own choice. On the contrary, he had entertained a strong repugnance to it. But having at length resolved to be a physician, he immediately brought all the energies of his mind to bear upon the one point, of thoroughly qualifying himself for an elevated position in the science and practice of medicine. His decision of character led him at once to impose proper limits on his favourite studies in general literature and the fine arts; in which, too, he had made considerable proficiency. He soon, however, had occasion to apply his skill as a draughtsman and colourist to the illustration of interesting subjects in pathology; and thus, by the skill and fidelity of his pencil, as well as by his close observation of disease, he not only contributed largely to the advancement of science, but also laid a deep and broad foundation for future eminence and usefulness. All the drawings iu his plates of morbid anatomy were executed by his own hand.

Nature also seemed to have peculiarly qualified Dr. Hope for auscultation. His hearing, as well as his vision, was remarkably acute. With an exquisite ear for music he could distinguish the slightest differences in sound, and could distinctly perceive sounds which were so faint as to be inaudible to most other persons. This important faculty, aided by a long-continued habit of closely observing the *general* indications of disease, eventually raised him to the highest distinction as an auscultator.

With such endowments and such principles of action, it will be readily supposed that Dr. Hope's career of academical studies must have given a high promise of professional excellence; and such was the fact. The same studious habits were uniformly continued during the whole period of actual practice. He was constantly accumulating facts and educing principles. On settling in London, having only *one* private acquaintance, his professional efforts for a few years were attended with scarcely any pecuniary advantage; yet was he never disheartened, but persevered with cheerful alacrity and untiring diligence in adding to his stores of practical information; investigating doubtful points; submitting theories to the test of rigid induction; and more especially, proceeding steadily with his two great works on the Diseases of the Heart, and on Morbid Anatomy. When the first of these was published, about nine years since, the whole profession united in commendation of its excellence; and, in the enlarged and improved form in which the author was fortunately enabled to reproduce it, in a third edition, two years before his death, it is now universally acknowledged to be the best book on the subject in any language. Dr. Hope became at once a high authority in that class of maladies. His brethren and the public flocked to him; and had he not prematurely broken down under the pressure of excessive labour, an ample fortune and the highest professional elevation, would in all probability have been his reward.

Dr. Hope's demeanor in the sick chamber was peculiarly appropriate. A kind, attentive interest, combined with a calm, will-sustained investigation of the case, secured his patient's confidence and regard.

As a lecturer, Dr. Hope was highly prized. A familiar acquaintance with his subject, and an evident desire to impart to his pupils, in the clearest and most impressive manner, the extensive information he possessed, were advantageously associated with a grateful fluency of diction, and considerable power of lively illustration. A high-toned morality, and an almost affectionate interest in the welfare of his class, inspired and obtained their high respect and attachment.

It is most gratifying to record that the celebrity thus acquired was borne with perfect equanimity. Dr. Hope continued the same modest, ingenuous, benevolent character as he had ever been. No assumption of consequence, no harshness of manner, no unkindness to junior practitioners or to the sick, whether rich or poor, ever derogated from his well-earned fame.

Dr. Hope's career was indeed a short one. At the age of forty, in the very zenith of prosperity, in the midst of honours and usefulness, with a rapidly increasing fortune, and in the full enjoyment of domestic happiness, he was called to resign them all. But futurity was not a blank to him; he had a hope full of immortality.

Our limits have necessarily excluded almost every kind of proof and illustration. Both, however, may be expected from the rich and ample materials which are already in a course of preparation for a more extended memoir.—*Ibid.*

---

*Establishment of Hospitals in China.*—In an early number of this Journal, we noticed the establishment of Medical Missions in China; and we are much gratified to learn that they have been most successful in their double capacity of providing for the temporal and spiritual wants of the singular people on the borders of whose vast country they are planted. One of the most zealous and enlightened of these excellent missionaries, the Rev. Peter Parker, M. D., of the U. States, is now in England for the purpose of raising "a permanent fund for the support of the 'Medical Missionary Society in China,' for the maintenance of the hospitals already established, and for the founding of others at every accessible and eligible part of China." The efforts to benefit the Chinese in this way in modern times are, we are told, briefly these: Alexander Pierson, Esq., surgeon to the Honourable East India Company, introduced successfully the art of vaccination in 1805; this has since extended widely through the empire. Dr. Livingston and Rev. Dr. Morrison opened an infirmary for the poor Chinese at Macao in 1820, which was sustained for some time, and alleviated much suffering. In 1827, T. R. Colledge, Esq., surgeon to the Honourable East India Company, opened his Eye Infirmary at Macao, and, during the three years of its continuance, afforded relief to no less than 4000 patients, among whom were persons in different ranks, and from various parts of the empire, from whom he received many and unequivocal tokens of gratitude. The Ophthalmic Hospital at Canton was opened by the Rev. P. Parker, M. D., October 1835, and the General Hospital at Macao, in July 1838. Up to the 17th June, 1840, these institutions had received upwards of 8000 patients, embracing every variety of disease. It was after long effort that a place was found for an hospital; and when at length a suitable building was rented, and previous notice had been given, the first day no patients ventured to come; the second, a solitary female affected with glaucoma came; the third day, half a dozen; and soon they came in crowds. Since then patients from all parts of the empire have availed themselves of the benefits of the hospital; persons of all ranks, military, naval, and civil officers, the Nanhæhëer, or district magistrate, the custom-house officer, salt inspectors, provincial judges, provincial treasurer, a Tartar general, governors of provinces, Commissioner Lin himself, and even a member of the imperial family.

The following extract from the same pamphlet which has supplied us with the above information respecting hospitals in China,* and which we recommend to the notice of our readers, points out, in a few words, the nature of the noble cause in which this gentleman is engaged, and which cannot fail to commend itself to every philanthropist.

"That the union of the art of healing with that of teaching, in the missionary of modern times, is as important as in the early ages of Christianity, is no longer doubtful. The experiment has been made, and succeeds. Healing by miraculous agency was employed at the commencement of the Christian era, chiefly as other preternatural powers were to establish the divinity of Christianity. A still further object was to exhibit the beneficial spirit of the gospel. The age of miracles and the occasion for them ceased together; but the *spirit* of the gospel is the same in every age. Healing the sick, opening the eyes of the blind, and the ears of the deaf, and causing the tongue of the dumb to speak, and the lame to walk, by natural and scientific means, is not less calculated, in the nature of things, to conciliate favour, and to demonstrate the disinterested and benevolent genius of Christianity now, than it

---

* "Statements respecting Hospitals in China," by the Rev. Peter Parker, M. D.—London, 1841.

was eighteen centuries ago. Though the practice of medicine and surgery among western nations is founded upon *science*, yet, to an uncivilized and superstitious nation, it has much of the appearance of a superhuman power, which may lawfully subserve a good end, if the truth of the case be distinctly stated, and their credulity be not imposed upon. The gratuitous practice of medicine and surgery, founding hospitals and infirmaries, confer a direct and great good upon suffering humanity. This species of charity is peculiarly needed in China. To sustain the hospitals already established in the empire, and to multiply them as the way is prepared, and in them to train up Chinese youth, to extend the blessings beyond the limits to which the policy of the government restricts foreigners, and to give a correct and scientific practice of medicine and surgery to an empire which exceeds in territory and population any other nation, is of itself a grand enterprise. The undertaking so great, is also *practicable.* Had the object no claims beyond those already alluded to, it would be deemed sufficient in respect to any christianised country; but in relation to China, they are the subordinate claims, compared with still higher ones, to which they conduct. In exhibiting the utility and importance of this object, let it not be supposed that any other is displaced. It is not to be lost sight of for a moment, that *Divine truth* is the great agent through which our ultimate aim is to be gained. While by the needle of the oculist the light may be poured upon the eye long dark, by the surgeon's knife the useless limb amputated, and by the physician's skill even the malignant disease may be cured, nothing short of a higher power can, in a moral sense, remove the film from the eye, clarify the spiritual vision, and heal the malady of sin. At present, however, it is but to a limited degree that the higher means can be employed; but to exhibit the spirit and the fruits of the gospel, Providence has remarkably opened the way; and it is fondly hoped that at no distant day, the Chinese will regard these benevolent offices as such, and permit us to publish and enforce the *precepts* of Christianity. After several years' experience and residence in China, the firm belief is expressed that in the present state of the Chinese, who are prejudiced against foreigners, as avaricious and barbarous, and possessed of no redeeming qualities, there is no method so directly adapted to remove false impressions, and to convince them of the true character of Christian men and the Christian religion, as by the plan adopted by the Medical Missionary Society in China."

We earnestly commend the sacred cause in which Dr. Parker and his brethren are engaged to the attention of all whose position and means enable them to promote it. Independently of the richer fruits of their heroic labours, we look forward, with confidence, to great benefits which medicine may expect from them, in the observation of new forms of disease, and in the discovery of new therapeutic means in the natural productions of this vast and unknown country, and amid the mountains of pharmaceutic rubbish which have been accumulating in their traditionary and written records, from periods anterior to all occidental history. We shall even hope for no inconsiderable addition to our stock of oriental medicine, from the work which Dr. Parker is now preparing for the press, founded on his observations and practice in China.—*Ibid.*

---

*On the Anastomoses of Nerves.* By A. W. Volkmann.—Under this name the author includes those cases in which the branch of one nerve is connected with the trunk of another, and passes in the latter, not downwards but upwards, and towards the nervous centre; an arrangement similar to that of the terminal loops, but occurring in these cases, not in isolated fibres, but in whole branches.

One loop of this kind exists frequently, if not regularly, between the fourth pair of nerves and the first division of the fifth in the calf. Looking at it with the naked eye, in one case the loop appeared to consist of a single filament; but, on examining it with the microscope, five branches were found to arise from it, of which four received their fibres from the fourth, and only one from the fifth. This last was fully ten times smaller than that part of the loop which was connected with the central portion of the fifth. If, therefore, this portion were considered as coming from the fifth, there would be only one-tenth of the fibres going to the periphery, while nine-tenths must have been passing to the brain within the sheath of the fourth nerve. For, since the fourth above the loop gave off no branches, there was no opportunity for these fibres to turn round out of it again, and pass to the periphery. The character of the loop here assumed can therefore be doubted only by supposing that the portion which seemed to come from the fifth, was really a branch passing into it; in which case it must have consisted of fibres of the fourth passing centripetally in the fifth. Such fibres might thus pass to the Gesserian ganglion, and thence go peripherally in some other branch of the fifth pair.

A second loop of the same kind appears to exist pretty generally in mammalia, between the second or third cervical nerve and the accessory. The author has found it in men, horses, dogs, calves, and cats. In the calf, the anterior branch of the second cervical gives off a considerable branch, which goes to the deep main branch of the accessorius. One portion of its fibres passes within the accessorius, towards the periphery; the other portion, centripetally. Above their union, branches are given off from the accessorius, so that there is an opportunity for the centripetal fibres just mentioned to pass out of it again, and proceed centrifugally; but the microscope shows that they do not do this. Or, again, as the vagus and the accessorius are connected at the ganglion on the former, the fibres in question might go into the vagus, and then proceed peripherally in it; but, in the first place, the author once found the loop where it was evident that the vagus and accessorius only lay close by each other, without mixing fibres; and, in the second place, he was fortunate enough on one occasion to trace a faciculus of fibres from the loop of the cervical completely into the root of the accessorius without a break. With the microscope and compressorium, he convinced himself that the fibres thus actually passed through the accessorius into the brain. Unless, therefore, it were admitted that the anastomosis between the accessorius and the second cervical connects two portions of the central organ, there remains only the hypothesis, that the fibres of this loop belonged to the accessorius, and they ran at first centrally in the cervical nerves, but at one of its branches turned round, and proceeded centrifugally. But experiments proved (as will be shown) that at least a portion of the fibres were sensitive, and came from the spinal cord.

A third and similar loop is found between the cervical nerves and the descending branches of the hypoglossal. In man, as well as in dogs, rabbits, sheep, lynxes, calves, and cats, connecting branches of the cervical nerves pass into the descendens noni, and in it some of their fibres proceed peripherally, but a larger portion of them go towards the nervous centre. These last fibres pass on to the tongue,* and it is remarkable that what is called the descendens noni appears to contain only a few fibres from the ninth nerve. Excluding those fibres in the descendens noni, which, as they go to the tongue, cannot be supposed to come from the ninth, there is the highest probability that many of the fibres which certainly do come from the ninth instead of remaining in the descending branch, go through a branch of the uppermost cervical nerves to the spinal cord.

The author believes that he has obtained a proof of this by dissection of the nerves in the sheep. In it the descendens noni, where it comes off, forms a plexus of numerous fibres, which, if it be carefully cleaned, and placed on a glass plate beneath the microscope, permits all its fibres to be closely traced. It is plain that, in this case, far more fibres pass to the descendens noni from the trunk of the ninth than it contains below the part where it is connected with the cervical nerves; and it thence follows, that a part of the fibres which pass from the ninth into its descending branch, do not remain in it, but go through the cervical to the cord; and this is the more probable, because these fibres could not again pass out of the cervical nerves to go peripherally, except at a very acute angle; and there are no signs of such an arrangement.

A fourth anastomosis exists between the second and third cervical nerves of the cat. The third, immediately at the origin of the anterior branch, gives off a large branch which first forms the loop already mentioned with the accessorius, but then passes completely into the second cervical nerve, in which a considerable portion of its fibres run backwards to the nervous centre. Between the first and second cervical nerves of the cat, there is also a constant communication, which, however, when closely examined, has rather the appearance of a plexus.

From what has been said, it results that there are nervous loops in which the fibres are placed together, but from which they do not go to be distributed in one organ. Such an arrangement must certainly be considered strange; but its strangeness is of the less weight against its reality, from its being exactly analogous to the terminal loops. The union of adjacent fibres (such as occurs in the terminal loops) is in no respect more intelligible than that of the fibres of distinct nerves; and, indeed, experiments on these larger loops may serve to illustrate the infinitely more delicate terminal loops, to which experimental investigation is inapplicable. Such experiments were therefore performed, and their results follow:

The skin was divided behind and below the transverse process of the atlas of a live calf, and the anterior branch of the second cervical nerve was exposed. It had already divided into two branches, of which one was directed backwards and downwards, the other forwards and downwards. The latter contained in a looser cellular sheath the finer branch, which lower down became free, and was connected with the accessorius. Around this anterior branch, an inch and a quarter below its exit from the vertebral column, a ligature was tied, producing to all appearance at the instant acute pain. A second ligature was then applied below the former, and therefore nearer to the accessorius. This second ligature was at first tied only gently, but the animal gave out manifest signs of pain. After a short pause, the knot was pulled quite tight, and again there was distinct evidence of pain, though the animal seemed to suffer less than when the first ligature, which was nearer to the origin of the cervical nerves, was applied.

The anastomosis between the cervical and the accessorius was exposed in a calf, and was found to consist of two separate filaments. On tying the first pain was produced, but a second ligature tied on it nearer to the accessorius excited no appearance of pain. Three ligatures were then placed one close behind another on the second branch. The first, which was put

* See paragraph 43, in the paper "On the Motor Influences of the Cerebral and Cervical Nerves."

in the middle of the branch, gave severe pain; the effect of the third, which was applied close to the union with the accessorius, was doubtful, the animal only drew back as if hurt. Supposing pain was produced, it must have been excited through branches of the accessorius, some of which other subsequent experiments proved to contain sensitive fibres.

The loop between the cervical and accessory nerves was exposed in a calf, and with short pauses after each application was tied three times. The first ligature excited pain, so did the second, which was placed nearer the spinal cord; but so did not the third, which was applied nearer to the accessorius than the first was. A fourth ligature was applied on the accessorius itself, and gave distinct pain.

The accessorius was exposed in a calf near its exit from the skull, and tied above its connexion with the second cervical nerve. The animal struggled and bleated during the drawing of the knot; after a pause the nerve was divided above the ligature, but there was no sign of pain. Then the divided nerve was repeatedly irritated at its peripheral end, and distinct indications of suffering were noticed every time.

Neglecting the very slight anomalies that were observed, and which may fairly be referred to the difficulty and tediousness of the operations, these experiments appear clearly to establish, 1st, that the anastomosis connecting the accessorius with the second cervical nerve is sensitive; 2d, that the course of conduction of the nervous influence is twofold, stimuli producing sensation by acting towards the brain as well as towards the spinal cord; 3d, the sensitive fibres in the loop that belong to the spinal cord are considerably predominant.

The anastomosis between the second and third cervical nerves in a cat was exposed. A ligature tied in the middle of the loop excited the severest pain. A second ligature applied close to the part where the loop seemed to come out from the second cervical nerve produced no reaction. A third placed close to the third nerve gave acute pain. The same experiment was repeated with the same result in another cat. In a third the loop was divided close to the second nerve, and on irritating the portion of it nearest to the third nerve, there were some some signs of pain, but the animal was too much exhausted to draw any strict deductions from its appearance of suffering.

[Other experiments were tried with the other anastomoses, but the author confesses that the difficulties of tracing such minute fibres as compose these nerves in the living subject, were too great to permit his attaining any definite result. He then enters upon a theoretical consideration of the intentions served by these anastomoses; but into this, as his hypothesis seems very far less valuable than his facts, it does not appear necessary to follow him.]—*Ibid.*, *from Müller's Archiv*, 1840. *Heft* v. *p.* 510.

---

*On the motor influences of the cerebral and cervical Nerves.* By A. W. Volkmann.—In experiments on the motor influences of the cerebral nerves, it is necessary to irritate their roots within the skull, because connexions take place between their branches soon after their exit from it, and sometimes even within its cavity. For this purpose the author adopted the plan of making longitudinal sections of the heads of fresh-slain animals, and irritating the nerves within the skull, either mechanically, or with the wires of a galvanic battery of which he could regulate the force. [We need not detail the cautions adopted to shut out all sources of error; the reputation which Volkmann has already earned in this branch of physiology is the best guarantee of the correctness of his experiments, and of the care with which he would draw results from them.]

I. *Of the oculo-motor or third pair of nerves.* 1. In a fresh-slain calf, the orbit was broken into and the muscles of the eye exposed. On irritating the root of the third nerve within the skull, contractions ensued in the levator palpebræ, rectus superior, rectus internus, rectus externus, obliquus inferior, obliquus superior, and retractor bulbi.* Repeated experiments on several calves, a cat and a sheep, always gave the same results. In the sheep the experiment was performed so as to remove all suspicion that the motions of the obliquus superior and rectus externus were secondary. The orbit was broken into, and the eyeball with all its muscles, except these two, removed; but the irritation of the oculo-motor nerve still excited contractions in them both, though in the obliquus superior much more violently than in the rectus externus.

2. A dog's skull was sawn through longitudinally, the orbit broken into, and all the recti muscles cut away directly after death. On irritating the third nerve, the direction of the pupil was not in the least altered; the eye did not move upwards, but it turned round on its axis just like a wheel on its axle-tree. The upper portion of the sclerotica described a small portion of a circular arc, and approached the outer angle of the eyelids. This experiment was repeated with a slight modification in another eye. The lower eyelid, and a portion of the lower margin of the orbit, were removed from a fresh-slain dog, the exposed obliquus inferior was irritated, and the result was the same; the rotation was like that of a wheel, and as regular as if the visual axis were so fixed as to prevent any other movement of the eye except this

---

* "My assistant has traced the branches of the third nerve to these last two muscles. The third in the calf gives a branch to the fourth nerve."

of rotation. The experiment was repeated and confirmed in the calf.

3. The healthy eye of a live calf was observed, and Bell's idea that at every wincing the pupil suddenly moves upwards and inwards, was found as little confirmed here as it is by examining the eyes of the rabbit, horse, and cat. A rotatory motion, however, was often noticed; the elliptical iris, with its long diameter, going almost transversely across the eye, moved round the pupil like the beam of a balance round its pivot, so that the posterior (external) end of the ellipse was considerably depressed, and the anterior as much elevated. The obliquus inferior was now exposed and divided, and the balance-like motion of the iris was no longer perceptible. When the conjunctiva was irritated, there was often no motion of the eyeball; sometimes, however, there was a sudden retraction of it, and sometimes a twitching of it upwards.

II. *Fourth pair.* 4. In a fresh-slain calf prepared as before, the fourth nerve was irritated within the skull. The eye turned round on its axis, and the balance-movement of the iris was manifest, but its direction was the reverse of that in exp. 3, the anterior part being depressed and the posterior raised. The pupil was not directed outwards and downwards. In the cat the same phenomena occurred, but in a less degree.

5. The orbit of a fresh-slain animal was broken into, and the fourth nerve galvanized. The exposed obliquus superior contracted, but it did so in like manner when the third nerve was irritated (exp. 1.) Endeavours to make other muscles move through the medium of the fourth nerve were uniformly unsuccessful.

III. *Sixth pair.* 6. On irritating this nerve after similar preparation of the head and orbit, violent contractions ensued in the exposed rectus externus, drawing the eye outwards, and producing various other twitchings. After removing this muscle, and irritating the sixth nerve, there ensued twitching movements of the eyeball, and the pupil was drawn into various directions. After removing all the other muscles, the retractor bulbi was found acting as often as the nerve was irritated; and according as one or other of its fasciculi contracted, the pupil was drawn into this or that direction. In no experiment could the inferior oblique be excited through the sixth nerve.

7. In another similar case irritating the sixth nerve excited irregular motions in different directions, and especially a turning of the pupil outwards; and every time the nerve was irritated, the third eyelid was moved from the inner towards the outer angle of the eye.

IV. *Fifth pair.** 10. In a fresh-slain calf, the smaller root, of this nerve was galvanized, and violent motions of the jaws ensued. The same result was obtained in other animals.—When the great root of this nerve, or the root of any other of the cerebral nerves was irritated, no motions of the jaw took place. The muscles of the lower jaw of a calf were exposed, and irritation of the small portion of the fifth nerve was then seen to produce contractions in the mylohyoideus, anterior portion of the digastricus, temporal, masseter, and internal pterygoid. The external pterygoid could never be exposed while the irritability lasted. On irritating the small portion of the fifth, neither the buccinator nor the soft palate was ever moved; the author believes, therefore, that though Bell, on anatomical grounds, attributed to this nerve the motions of the buccinator muscle, and Valentin on similar evidence those of the soft palate, its motor influence is really limited to the muscles enumerated above.

V. *Seventh pair, or facial.* 11. In calves, dogs, sheep, and goats, the root of the facial being exposed and galvanized, distinct contractions occurred in the following muscles; frontalis, buccinator, orbicular palpebræ, orbicularis oris, part of a muscle moving the nose, and of another moving the angle of the mouth, the attrahens, retrahens, and attollens auris, the posterior belly of the digastricus, the stylo-hyoideus and platysma.

12. The author could never succeed in exposing the muscles of the tympanum so as to see them act, but in his attempts he several times found irritation of the chorda tympani produce contractions of the buccinator.

13. On irritating the portio intermedia Wrisbergi, twitchings ensued in all the muscles of the face and ear, in the same manner as if the great portion of the facial had been irritated.

14. In a great number of experiments, irritation of the facial never produced motions of the tongue. 15. Neither is the soft palate ever moved by the influence of the facial, either in sneezing, as Bidder supposed, or in swallowing, as Valentin held. In the cases in which such motions seem to occur, the movements of the palate are only secondary to those of the tongue. The facial governs the digastricus and stylo-hyoideus; and when these are divided, or their action has ceased, the motions of the soft palate are no longer discernible, nor can they ever be excited, though all the muscles of the face and ears are thrown into active contractions. It is therefore manifest that the branch of the facial, which, under the name of superior vidian, passes to the sphino-palatino ganglion, does not convey any motor filaments to the soft palate.

VI. *Glosso-pharyngeal.* 16. This nerve arises in the calf, cat, and man, by two distinct roots, each of which may be separated into two fasciculi. On the larger root (and often within the skull) lies the Ehrenritter ganglion. But this does not seem to be really distinct from

* For convenience of reference, the numbers of the paragraphs are marked as in the original paper; a considerable portion on the action of the muscles of the nerve is here omitted, but is printed in a separate extract.

the petrous ganglion; for often only one ganglion can be found, and when there appear to be two, they are always connected by a tract of ganglionic substance. The separation of the two is therefore accidental; and however varied their apparent arrangement, it may be stated as a general rule, that all the fibres of the glosso-pharyngeal nerve pass through ganglionic matter. It is impossible therefore to compare the two roots of this nerve with those of the spinal nerves, or the Ehrenritter ganglion with the ganglia of the spinal sensitive roots.

17. After numerous unsuccessful attempts to produce motion by irritating this nerve, the author met with a case in which its two roots were separated, not only from the vagus, but from each other by dura mater. Irritation of the larger of these roots produced no effect, but that of the smaller (which in the previous experiments had been overlooked) excited violent motions in the pharynx. On exposing the latter it appeared that the middle constrictor and the stylo-pharyngeus were acting. This result was confirmed on two calves and two cats, so as fairly to establish, 1st, that the glosso-pharyngeal nerves govern only these muscles; 2d, that no other nerve has any influence on them; and 3d, that only the small root of the nerve is endowed with motor power. In the experiments of Valentin and John Reid, who could find no direct motion produced by irritating the glosso-pharyngeal, its smaller root must have been overlooked, as it was by the author in his first experiments.

VII. *Nervus vagus.* 18. In the dog and sheep a part of the roots of the vagus pass by the ganglion, and are connected with it only by cellular tissue; but in the calf this is not the case; several fibres seem indeed to go behind the ganglion, but on these there are minute irregularities, which when examined with the microscope are found to be true ganglionic matter. All the fibres in the calf therefore pass through ganglionic matter; a fact which is of some importance in relation to the following experiments, and to the physiology of ganglia and gangliated nerves.

19. A fresh calf's head was separated from the trunk and sawn longitudinally, so that the soft palate and upper part of the pharynx could be seen. The medulla oblongata was then drawn aside, and the roots of the nervus accessorius were carefully divided with scissors.—No motions took place. The roots of the vagus were then cut, separately and singly from behind forwards; and at almost every section motion took place, either in the soft palate, or in the upper part of the pharynx, or in both at once. The experiment was repeated with every caution on five animals, and always gave the same results.

20. A calf's head was sawn longitudinally, and several roots, supposed to belong to the vagus being irritated, motions were produced in the pharynx and soft palate. Around each root a silk thread was tied, and when the experiment was finished they were all traced to their origins. By far the greater part belonged to the vagus; one pair only came from the glosso-pharyngeus; but as experiment 17 shows that the latter nerve can excite motion in neither the upper part of the pharynx nor in the soft palate, it follows that the motions that were seen in this case must have depended on the vagus.

21. In another calf's head similarly prepared, the accessorius and glosso-pharyngeus were carefully removed as far as the foramen lacerum. The roots of the vagus were then galvanized, and motions took place in the superior and inferior constrictors of the pharynx, and in the crico-thyroideus, and in all the muscles of the soft palate. Subsequent dissection showed, that this experiment had been correctly performed.

22. Heads of calves, sheep, goats, cats, and dogs, were prepared directly after death, so as to expose the muscles of the larynx, pharynx, and soft palate. The roots of the vagus were then galvanized, and independent contractions ensued in the levator palati, azygos uvulæ, constrictor superior, c. inferior (but never in the c. medius,) pharyngo-palatinus, and crico-thyroideus.

23. Different calves, sheep, goats, dogs, and cats were killed, by opening the carotids or abdominal aorta, and the descending branches of the vagus were exposed. Its roots were then irritated within the skull, and the most violent motions of the œsophagus instantly ensued, extending through its whole length, and exactly resembling those of voluntary muscles. The stomach never moved except at its cardiac portion by the communication of the movements of the œsophagus.

24. The larynx viewed from the spine during these experiments often presented manifest motion, but more rarely an alteration in the dimensions of the glottis, than a twitching of the prominent parts of the arytenoid cartilages. When the muscles were exposed the crico-arytenoideus posterius and lateralis were seen to move in the cat and calf, and the latter only in the dog; but the results of these experiments were often confused by the nerves being divided in the process of exposing the muscles; and it was probably on this account that the arytenoid muscles did not contract on irritating either the vagus or the accessorius.

25. After a cat had been killed by a stab in the heart, the vagi were divided below the superior laryngeal, and the glottis were exposed. Irritation of the superior laryngeal did not move the glottis, but on irritating the lower part of the nerve the glottis was widened, and sometimes the vocal ligaments were stretched. On irritating the superior laryngeal the exposed muscles that contracted were the constrictor faucium superior, the crico-thyroideus, and (in dogs and calves,) the thyro-hyoideus. On irritating the lower part of the vagus, the muscles that contracted were the crico-arytenoideus posticus and lateralis.

26. In two young dogs the cerebrum and cerebellum were removed ; the glottis being exposed, it opened and shut with every effort of respiration. In one, the superior laryngeal nerves were divided, aud the motions of the glottis were not in the least affected in consequence. In the other the vagi were divided, so as to destroy the influence of the recurrent, and the glottis remained permanently closed. The same result followed the division of the vagus within the skull in young dogs ; but it is probable that this closure of the glottis is the consequence, not of any muscular contraction, but of the pressure of the atmosphere, which, when the muscles are paralyzed, forces the edges of the glottis together at every inspiration.

28. In different animals killed by bleeding, the chest and abdomen were opened, and the roots of the vagus galvanized, but no motion was seen in either stomach, intestines, heart, or trachea ; or, at least, the increase of the peristaltic motions of the first was not more than would result from the contact of the air, or from the communication of the movements of the œsophagus.

29. No one of the muscles just mentioned as being influenced by the vagus could be made to contract through any other nerve. In experiments on nearly 100 animals, there were but two which seemed to indicate that the influence ascribed to the vagus belonged to other nerves ; in one cat irritation of the accessorius excited motion of the soft palate, and in one calf that of the glosso-pharyngeus excited contractions of the crico-thyroideus, but it is quite possible that these anomalies were the results of error in the experiments.

VIII. *Nervus accessorius.* 30. When this nerve was galvanized within the skull in cats, dogs, goats, calves, and rabbits, violent motions ensued in the neck and shoulder, and when the skin was removed the muscles contracting were seen to be the whole of the sterno-cleido-mastoid and the trapezius ; no motion was ever seen in the soft palate, (except in the one cat in No. 29,) the pharynx or the larynx, but they were acted on as soon as the vagus was irritated.

31. The heart was exposed in a rabbit killed by hæmorrhage ; its contractions were but few and distant, and they did not in any degree correspond with the repeated irritation of the accessorius within the skull.

All these results occurred uniformly in a great number of experiments ; the muscles of the larynx, pharynx, and soft palate, &c. were always moved when the vagus, but never when the accessorius was irritated ; the excitement of the latter produced motions only in the trapezius and sterno-mastoid. Now since it is not at all probable, that in irritating the accessorius certain roots belonging to it were *always* missed, and that in irritating the vagus these same fibres were *always* included, there can, Volkmann thinks, be no doubt that the motor functions he has ascribed to the vagus do really belong to it, and not to the accessorius, and that the latter has no influence on the motions of the palate, fauces, and glottis.

IX. *Hypoglossal Nerve.* 34. This nerve always arises in several bundles of roots that pass through separate holes in the dura mater; and a ganglion being usually seated on one of them, has given rise to the general opinion that this is a nerve of mixed function. The ramus descendens has always been considered a branch of this nerve, but if the mode in which they are connected be examined, it will be found that all the fibres of the former do not pass centripetally into the trunk of the hypoglossal, but that some go on through it centrifugally, and therefore cannot be said to come from it. This is the case in man, calves, sheep, rabbits, and cats. In the horse, the author once saw that what is commonly called the descendens noni was really a ramus ascendens, receiving no fibres from the hypoglossal, but conveying all the fibres it contained to it. These fibres came from the two upper cervical nerves.—[See further the paper on Anastomoses of Nerves.]

35. In several fresh slain animals the hypoglossal was irritated at its root ; motions ensued in the styloglossus, hyoglossus, genioglossus, lingualis, and (in the calf) the hyoepiglottis. In rabits, irritation of either root excited contraction. Experiments were repeatedly made to see whether irritation of this nerve would produce motions of the omo-hyoideus, sterno-hyoideus, and sterno-thyroideus, but only the sterno-hyoideus could be made to move, and this in only two calves and one dog out of all the animals experimented on ; facts which, with those mentioned in the preceding paragraph, are sufficient to prove that the hypoglossal gives but a few motor fibres to the descendens noni, and in general none but those for the thyro-hyoideus.

36. Each root of the hypoglossal was separately divided in a calf, and at every division a distinct motion of the tongue occurred. The little root with the ganglion however was overlooked ; but in subsequent experiments, on galvanizing it, motion was repeatedly and regularly excited in a small space on the back of the tongue. The same experiment was twice repeated with every caution lest the galvanic action should be too powerful, but the result was in both the same.

X. *The cervical nerves.* 37. These join the cerebral nerves in two different modes, their fibres pass into them either centrifugally or centripetally. The latter direction is generally disregarded, but repeated microscopic examinations have proved to the author, that branches of cervical nerves do thus pass to parts where they are least expected to be found. (This subject is treated of in a separate paper, on Anastomoses of Nerves.)

38. A sheep having been pithed between the occiput and atlas, the pharynx was exposed, and a galvanic current directed through the cervical part of the cord. Violent contractions ensued in the muscles of the neck, but none in the œsophagus, and this occurred repeatedly with very slight galvanic forces. There was indeed a slight undulatory motion of the œsophagus, such as the contact of the air might cause, but no general muscular action corresponding with the stimulus, or like that produced by irritating the vagus (23.)—The same results followed irritation of the cervical nerves themselves where they are leaving the vertebral canal.

39. Irritation of the (motor) roots of the first cervical nerve excited motion in the sterno-hyoideus, sterno-thyroideus, and thyro-hyoideus (which was in one case moved by irritation of the hypoglossal also,) but neither the sterno-mastoid nor the pharynx. Irritation of the second nerve excited several muscles in the neck, but none of those just mentioned, nor the œsophagus.

A number of similar experiments all tended to prove negatively that the movements of the pharynx and œsophagus are independent of the cervical nerves and governed by the vagi.

43. The muscles of the tongue were exposed in a fresh-slain sheep, and the descendens noni divided. At the instant of division the genio-hyoideus twitched, and this was repeated at every irritation. The central portion of the nerve was galvanized in several animals, and motions ensued in the hyo-glossus, genio-hyoideus, genio-glossus and lingualis, but it was difficult to say whether any of these, except the first, were moved primarily. Irritation of the root of the first cervical nerve regularly produced a motion of the tongue by which it was curved upwards, the lingualis being set in action.

45. As the general results of these experiments (which however are probably more valuable in their details,) the author gives the following conclusions :

A. All the nerves of the head, except the three of special sense, are motor (in a greater or less degree ?)

B. Each muscle of the head (two of those of the eye excepted) receives its motor power from one nerve only, and therefore the voluntary and automatic motions of these muscles both depend on the same nerve.

C. This was found true of so many animals, that it is probably true of man also.

D. Some muscles of the tongue receive (as Valentin showed the iris does) branches from the cervical as well as from the cerebral nerves, a fact by which the defects of speech in disease of the cord are intelligible.

E. Motor nerves may have ganglia on their roots.—*Ibid., from Müller's Archiv.*

---

*Report of the lateral operations for stone in Naples, with Statistics of Lithotomy in the last ten years.* By M. S. de Renzi.—*First series.*—32 men and 1 female operated upon in the year 1839.

Among these 33 patients 10 were under 15 years of age, 22 were adults, 1 was aged. In all there was but a single calculus; 24 were operated on and cured, 9 died. Five were cured in 1 month, 8 in 40 days, 5 in 2 months, 6 in 3 months. Hemorrhage occurred in 5 cases. In 10 the most severe symptoms manifested themselves. In 2 there had been a species of "*pourriture d'hopital.*" Of the 9 who died, 4 had very large stones, which rendered long and painful manipulations necessary. Of these, 2 died on the second day, 1 on the eighth, 1 on the thirteenth, and all with the symptoms of enteritis or cystitis. Of the other 5, in 1 the dilated bladder filled the pelvis, adhering to the soft parts and the psoas muscle, with an effusion of sanious pus in its cavity. Another, aged 48 years, had purulent collections in the bladder. A child died of vermicular enteritis the third day after operation; 2 others survived 9 and 12 days; and both had purulent collections in the kidneys.

*Second series*—14 men, all operated on with success, 5 of whom were under 15, all the other adults. None of them had dangerous symptomps. In two-thirds of these cases the stone was of a moderate size, in the other third it was large.

*Statistics of Lithotomy, from* 1821 *to* 1828, *and during* 1829.

1821 to 1828.—Men, 579; Women, 17 Cured, 503; Died, 91; Children, 306; Adults, 231; Old Men, 59.

1821 to 1839.—Men, 46; Women, 15; Cured, 38; Died, 9; Children, 15; Adults, 31; Old Men, 1.—*Il Filiatre Sebezio.*

---

*On the present mode of treating the Itch at Berlin.* By Dr. Hauck.—The treatment of itch has lately been made the subject of extensive experimental observation in the Berlin hospitals, and it is satisfactory to learn that a slight modification of what is termed the English method, has been found in every respect superior to any other that was adopted, accomplishing as it does all the desirable objects of curing the disease quickly, certainly, and economically.

The remedy employed was the sulphur and and soap liniment of the Prussian Military Pharmacopœia, composed of one part of flowers of sulphur, and two parts of soap mixed with sufficient hot water to make them into a soft ointment. The patients after a warm bath of soap and water had been applied, were placed undressed in a chamber, kept constantly at a temperature of 96° Fah., and well rubbed with the ointment over all the parts where he eruption had appeared three times a day, and then made to sweat profusely by putting them into warm beds. This system was continued three days and nights; on the morning of the fourth each patient had a warm bath, and then if not cured, was provided with clean bed and body-linen, and put in a ward of ordinary temperature, in which the suspicious parts were still rubbed with the ointment, and a warm bath taken every other day. In general, no internal medicines were given; but the diet allowed was reduced to a fourth portion, and water only given them to drink.

In this manner, with but one short interval, 1981 were treated and cured between September, 1839, and February, 1840, making the total number of days of treatment 16,890, which gives on the average 8 days and a small fraction for the cure of each patient, and for the expense of each about two dollars. The exact result was, that

| | | |
|---|---|---|
| In 3 days there were cured | | 42 |
| —4 | „ „ | 161 |
| —5 | „ „ | 333 |
| —6 | „ „ | 376 |
| —7 | „ „ | 207 |
| more than 7 | „ „ | 859 |

The treatment of these last was prolonged by many circumstances which can hardly cast discredit on the remedies. In many among them the itch was soon cured, but they remained under treatment for the ulcers which had come on from long neglect of it, or were kept in the hospital till there was no chance of the ulers communicating the disease. Others among them after being cured of the skin-disease had to be treated for other affections, such as ophthalmia, fever, &c.; and others again had their cure delayed by an obstinate refusal to adopt all accessory treatment. And in addition to these causes giving rise to an apparent increase of the length of time necessary for the cure of the disease, there were some others dependent on the management of the hospitals, and other circumstances quite foreign to the treatment adopted, but which, had they not existed, would have permitted the average number of days of treatment to have been stated much lower.

In the whole 15 months there occurred only 8 cases of relapse, less that is than half per cent. of the cases treated; and among these there was, in many, good reason to suspect a fresh infection. The other cases in which there appeared to be a relapse were in fact only examples of eczema resulting from the stimulus of the skin by the sulphur. In no case did the treatment give rise to any general disorder, or to the inflammations and congestions which some have described as resulting from it.—*Ibid.*, *from Med. Zeit.*

---

*Notice respecting the operation of Stammering.*—"To prevent all questions of priority, I hereby inform my colleagues, that I first performed my operation for stammering on the lad Donau, thirteen years old, on the 17th of January, 1841, and had the honour to present him completely cured at the meeting of the Hufeland Medical Society, on the 22d of January, 1841. DIEFFENBACH."—*Ibid.*, *from Med. Zeit.*

